# Antioxidant Activity and In Vitro Antiglycation of the Fruit of* Spondias purpurea*

**DOI:** 10.1155/2018/5613704

**Published:** 2018-08-29

**Authors:** Alethia Muñiz, Efren Garcia, Daphne Gonzalez, Lizette Zuñiga

**Affiliations:** ^1^CONACYT-IPICYT/CIIDZA, Camino a la Presa de San José 2055, Col. Lomas 4 Sección, CP 78216, San Luis Potosí, SLP, Mexico; ^2^Laboratorio de Química Supramolecular y Nanociencias, Instituto Politécnico Nacional, Acueducto s/n, Barrio la Laguna Ticomán, CP 07340, Ciudad de México, Mexico; ^3^Facultad de Ciencias Químicas, Universidad Autónoma de San Luis Potosí, Av. Dr. Manuel Nava No. 6, Zona Universitaria, CP 78210, San Luis Potosí, SLP, Mexico

## Abstract

Hyperglycemia in diabetes mellitus causes irreversible life-threatening micro- and macrovascular complications. There is evidence that the glycation reaction leads to a chemical modification of the proteins contributing to the complications of diabetes. It is known that advanced glycation end products (AGEs) are formed by glycation and oxidation reactions called glycoxidation. CML, a nonfluorescent AGE, has become a biomarker of glycoxidative damage; other AGEs appear to induce oxidative stress, which results in cytotoxicity. To determine antioxidant activity, the FRAP, DPPH, and TEAC tests were used, as well as the polyphenols content using Folin-Ciocalteu's method. To evaluate the antiglycation activity, the BSA/glucose system was used, and the fructosamine concentration, protein carbonyl content, thiol, and CML groups were determined. The results obtained show that the hexane extract of the fruit of* Spondias purpurea* (CFH) effectively inhibits the glycation reaction, in addition to increasing the thiol groups and decreasing levels of fructosamine, protein carbonyl, and CML. In addition, CFH presented significant antioxidant activity. CFH inhibits the glycation reaction; therefore, it can help prevent complications related to AGEs in diabetes mellitus; it also reduces oxidative stress and is effective in protecting proteins from oxidative damage.

## 1. Introduction

Diabetes mellitus (DM) is an endocrine disorder characterized by the presence of hyperglycemia, resulting in chronic complications that affect the eyes, blood vessels, nerves, and kidneys. Hyperglycemia plays an important role in the pathogenesis of diabetic complications, as it increases protein glycation, as well as the progressive accumulation of advanced glycation end products (AGEs), which are complex structures that mostly produce fluorescence. AGEs inhibit the molecular conformation of proteins and alter enzyme activity [1]. The glycation of proteins and the formation of AGEs are a complex process influenced by different factors such as metal ions, *α*-dicarbonyls, and oxidative stress; the latter increases the formation of free radicals, which contribute to biomolecular damage in DM. The first compound that was studied both in vitro and in vivo to inhibit the formation of AGEs was aminoguanidine (AG); however, this presents toxic side effects when administered to diabetic patients and causes pernicious anemia [2]; also diabetic rats treated with AG showed pancreatic tumors [3]; therefore, it is very important to find new therapeutic compounds of natural origin that inhibit the glycation reaction due to the therapeutic potential they offer. In this regard, herbal medicines have been widely used since ancient times to treat different diseases, because plants have different compounds with various biological activities such as tannins, phenolic acids, flavonoids, terpenoids, alkaloids, and steroids [4].

*Spondias L*. (Anacardiaceae) is a genus of 17 species, which are found in different tropical regions of the world [5]; seven of them are neo-tropical and ten are from the Asian tropics. The Mexican plum (*Spondias purpurea)* has been consumed since pre-Hispanic times; it is distributed naturally along the western coast and the southeast of México [6] and has a low production cost since it grows spontaneously, adapting to poor and thin soils in which other crops do not grow, besides presenting resistance to drought by defoliation.

Due to this, the use of this species has been suggested as a strategy for agriculture in Mexico, where in the dry season no other fruit is produced [7]. Its distribution in Mexico is located from the Sonora coast to Chiapas and the Balsas basin in the south and includes the states of Yucatan, Quintana Roo, Northern Veracruz, and San Luis Potosí [7].

The Mexican plum is a round or ovoid drupe which has a size of 2 to 5 cm, a thin epicarp, which is smooth or semismooth and can turn reddish, yellow, or reddish brown when ripe [8]; in addition, its endocarp is thick and fibrous and its mesocarp has a pleasant taste and aroma.

The consumption of functional foods associated with the prevention of chronic degenerative diseases in recent years has been increasing; this beneficial effect is due to the presence of bioactive compounds with antioxidant properties, which act on harmful molecules such as free radicals, interacting and destabilizing proteins, nucleic acids, and lipids [9]. The antioxidant compounds have the capacity to eliminate the harmful effects of the reactive oxygen species (ROS), thus reducing the levels of cellular oxidative stress and preventing the generation of damage in important biocomposites. In addition, of the biological properties present, antioxidants of natural origin are also of interest in the fields of cosmetology, pharmacology, and the food industry [7].

In this regard, its fruit provides a high caloric density, vitamin C, and minerals such as potassium and calcium [10]; it also possesses phenolic compounds [11] such as tannins, phenolic acids, and flavonoids [12], which act as natural antioxidants, offering protection against chronic degenerative diseases [13] such as diabetes mellitus (DM).

Due to the fact that the glycation reaction, as well as the oxidation, are involved in the complications of diabetes, in the present investigation the effect of the hexane extract of the fruit* Spondias purpurea* was examined in the glycation reaction, as well as its antioxidant potential.

## 2. Materials and Methods

### 2.1. Obtaining the Extract

**Figure 1:**
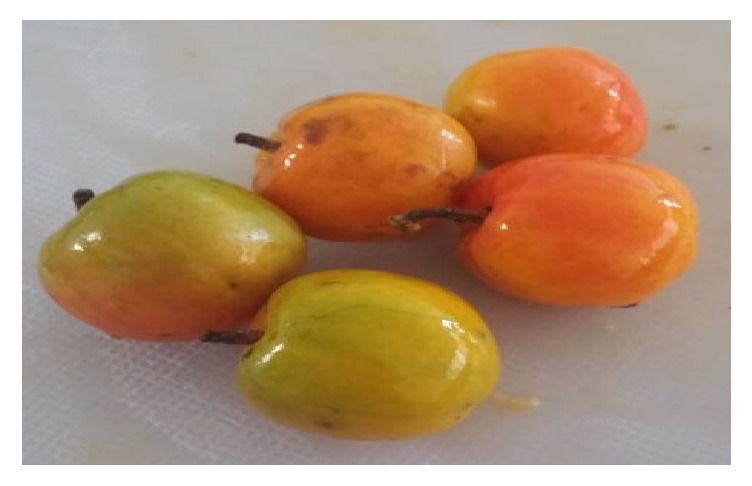
Mexican pump* (Spondias purpurea)*.

4 kg of Mexican plum* (Spondias purpurea)* was collected in Cuautla, State of Mexico, in the month of April of 2017, in a state of commercial ripeness, showing a yellow-reddish color; the size of the fruit was 3 to 4 cm (Figure 1). The fruit was dried in an air current stove; then the fruit was ground in a manual mill. Two hundred grams of dried and milled fruit was extracted with 1500ml of hexane using a soxhlet apparatus. The extract (CFH) was filtered and concentrated by rotary vacuum evaporator for complete removal of solvent.

### 2.2. Ferric Reducing Antioxidant Power (FRAP) Assay

To prepare the FRAP reagent, 2.5 ml of 10 mM tripyridyltriazine dissolved in 40 mM HCL, 2.5 ml of FeCl_3_, and 25 ml of acetate buffer 0.3 M pH 3.6. The extract was dissolved in phosphate buffer (1 mg / ml). 0.2 ml of the extract solution and 1.8 ml of the fresh FRAP reagent were mixed; then the sample was incubated at room temperature for 30 minutes. The increase in absorbance was measured at 595 nm (6405, JENWAY). The FRAP value was calculated by performing a standard curve, using FeSO_4_. The FRAP values were expressed as mM of FeSO_4_ per gram of dry extract [14].

### 2.3. DPPH Radical Scavenging Activity

To determine the activity of the DPPH radical, CFH was dissolved in a phosphate buffer pH 7.4; then 100 *μ*l of this solution which contained CFH dissolved in PBS (0.0125-0.100 mg / ml) was added and 100 *μ*l of a DPPH solution was added (0.2 mM); finally the solution was incubated at room temperature for 30 minutes, the absorbance was measured at 515 nm (6405, JENWAY), and ascorbic acid was used as control. The antioxidant activity was calculated by determining the absorbance at different concentrations [15].

### 2.4. Trolox Equivalent Antioxidant Capacity (TEAC) Assay

The method was carried out according to the method described by [16] with some modifications to generate the radical cation ABTS · +, 5ml of an aqueous solution of ABTS (7Mm) and 88*μ*l 140mM of potassium persulfate were added, and the solution was incubated at 29°C for 16 hours in the dark. Subsequently, 500 *μ*l of CFH dissolved in phosphate buffer was added to 990 *μ*l of ABTS·+. After 6 minutes the absorbance at 734 nm (6405, JENWAY) was measured. As a positive control, trolox was used, and the TEAC value was calculated from a standard curve using trolox. The values were expressed as mg of trolox per gram of dry extract.

### 2.5. In Vitro Glycation of Bovine Albumin

To determine the antiglycation activity, the method of [17] was followed with some modifications. The extract containing 0.02% sodium azide was incubated at 37°C, with BSA (10mg/ml) and 500mM of phosphate buffer for a final volume of 5 ml at pH 7.4, with a final concentration of 2mg/ml of BSA, 40 mM glucose, and 0.30-5mg/ml CFH dissolved in DMSO. Subsequently the solution was mixed and incubated for 1, 2, 3, and 4 weeks. As a positive inhibitor aminoguanidine was used, the fluorescence intensity was measured at 370 nm excitation and 440 nm emission (GENios, TECAN).

### 2.6. Fructosamine Determination

To determine the concentration of fructosamine, the NTB assay described by [18] was used with some modifications, for which, after 1, 2, 3, and 4 weeks of incubation, 10 *μ*l of glycated BSA was taken with 90 *μ*l of 0.5 mM NTB in 0.1 M carbonate buffer at pH 10.4, and at a temperature of 37°C. After 10 minutes the absorbance at 530 nm (6405, JENWAY) was read. As a standard, 1-deoxy-1-morpholino-fructose (1-DMF) was used.

### 2.7. Determination of Protein Carbonyl Content

To determine the carbonyl protein content in CFH, the method described by [19] was followed with some modifications, for this purpose after 1, 2, 3, and 4 weeks of incubation, 1 ml of 10mM 2,4-dinitrophenylhydrazine (DNPH) and 2M HCl was added to the glycated samples and incubated at room temperature for 30 minutes. After the mixture, 1 ml of cold TCA (10% w / v) was added and centrifuged at 3000 x g for 10 minutes. The sedimented protein was washed three times with 2 ml of ethanol/ethyl acetate (1: 1) dissolved in 1 ml of 6 M guanidine hydrochloride at pH 2.3. Finally, the absorbance was measured at 370 nm (6405, JENWAY). The molar extinction coefficient of the DNPH was used to calculate the carbonyl content (*ε*=2.2×10^4^ cm^−1^M^−1^). The results were expressed as nmol carbonyl/mg protein.

### 2.8. Determination of Thiol Group

After 1, 2, 3, and 4 weeks of incubation, the free thiol groups were determined in the glycated samples. We used the method described by Ellman with some modifications [20]. The glycated samples were incubated with 5 mM of 5,5′-dithiobis(2-nitrobenzoic acid) (DTNB) in 0.1M PBS at pH 7.4 for 15 minutes. Subsequently, the absorbance was read at 412 nm (6405, JENWAY); to calculate the concentration of free thiol a standard curve of L-cysteine was used. The results were expressed as nmol/mg of protein.

### 2.9. Determination of N*ε*-(Carboxymethyl) Lysine

At the end of the 4-week incubation, N*ε*-(carboxymethyl) lysine (CML), a major antigenic AGE structure, was determined by using enzyme linked immunosorbent assay (ELISA) kit. The concentration of CML was calculated by using the standard CML-BSA curve from the assay kit.

### 2.10. Statistical Analysis

The results were expressed as the mean ± standard error of the mean (SEM) (n = 3). The statistical significance of the results was evaluated by using one-way ANOVA. The least significant difference (LSD) test was used for mean comparisons, and p< 0.05 was considered to be statistically significant.

## 3. Results

### 3.1. In Vitro Glycation of Bovine Albumin

**Figure 2:**
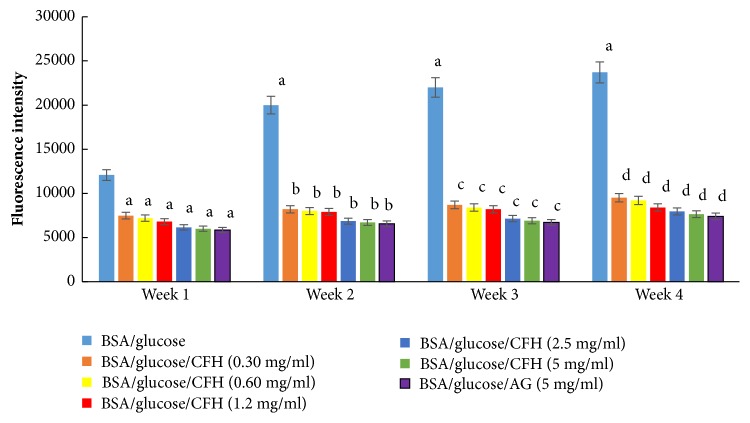
Effect on different concentrations of the hexane extract of* Spondias purpurea* (CFH), in the formation of fluorescent advanced glycation end products, using the BSA-glucose system during an incubation period of 1 to 4 weeks. Each value represents the mean ± SE (n = 3). ^a^p < 0.05 when compared to BSA/glucose at week 1; ^b^p < 0.05 when compared to BSA/glucose at week 2; ^c^p < 0.05 when compared to BSA/glucose at week 3; ^d^p < 0.05 when compared to BSA/glucose at week 4.

Figure 2 shows the fluorescence intensity for 1, 2, 3, and 4 weeks. By incubating BSA/glucose the fluorescence intensity increased significantly over the weeks. Regarding the experimental groups incubated with BSA/glucose/CFH at different concentrations, it was observed that the intensity of fluorescence is dependent on the concentration, since it decreased significantly during the four weeks that the experiment lasted. At the end of the test the BSA/glucose/CFH group at a concentration of 5 mg/ml showed a decrease of 67.73%, and the BSA/glucose/AG system showed a decrease in fluorescence intensity of 68.77% compared to the BSA glycated in the same time interval.

### 3.2. Determination of Fructosamine

The effect on fructosamine levels using CFH at different concentrations is shown in Table 1; significant differences between the different treatments were observed. In the BSA/glucose system the concentration of fructosamine increased significantly during the four weeks that the experiment lasted, showing a value of 88.38 mM at the end. However, when CFH was added to the BSA/glucose system, at different concentrations, fructosamine levels decreased significantly, highlighting the concentration of 5mg/ml at four weeks of incubation, which showed the greatest decrease obtaining a value of 80.74 mM, a value similar to that of the BSA/glucose/AG system (5 mg/ml), which showed a value of 79.59 in the same time interval.

### 3.3. Determination of Protein Carbonyl Content

Protein oxidation occurs during the glycation process, which is why the carbonyl protein and thiol groups were tested.  Table 2 shows the carbonyl protein content at different time intervals (1, 2, 3, and 4 weeks). The glycated BSA showed a significant increase as the weeks of the experiment passed; however, the BSA/glucose/CFH system significantly decreased protein oxidation, obtaining at the end of the test and at a concentration of 5 mg/ml a value of 80.49 nmol/mg in comparison with the glycated BSA which showed a value of 84.09 nmol/mg in the same time interval, while the BSA/glucose/AG system (5 mg/ml) showed a value of 78.38.

### 3.4. Thiol Group Estimation

The effect of CFH on the oxidation of thiol groups is shown in Table 3. The BSA/glucose system showed a significant decrease in the thiol groups during the four weeks that the experiment lasted. While using the BSA/glucose/CFH system at different concentrations thiol groups increased significantly, the concentration of 5 mg/ml after four weeks was the one that showed the highest increase in thiol groups, obtaining a value of 68.52 nmol/mg protein compared to the glycated BSA which had a value of 60.05 nmol/mg protein. When using the AG in the BSA/glucose system, it showed a value of 69.08 nmol/mg protein at the end of the experiment.

### 3.5. Determination of *N*^*ɛ*^-(Carboxymethyl)lysine (CML)

**Figure 3:**
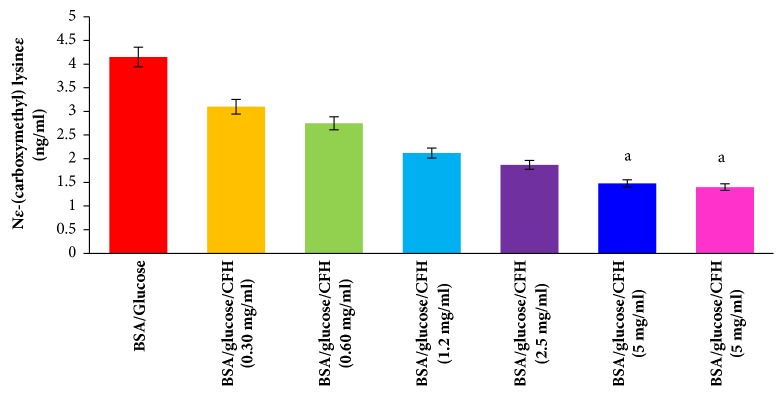
Effect to different concentrations of the hexane extract of* Spondias purpurea* (CFH) in the* N*^ɛ^*-(carboxymethyl)lysine *formation using the BSA-glucose model during an incubation period of 4 weeks. Each value represents the mean ± SEM (n = 3). ^a^p < 0.05 compared to BSA/ Glucose.

This test is used to determine nonfluorescent AGEs. In the BSA/glucose/CFH system at different concentrations (0.30-5 mg/ml), a concentration-dependent decrease in the CML concentration (Figure 3) could be observed at a concentration of 5 mg/ml of CFH a value of 1.48 ng/ml of CML, a value very similar to that obtained in the BSA/glucose/AG system, which showed 1.40 ng/ml; in contrast the glycated BSA had a CML concentration of 4.15 ng/ml.

### 3.6. Antioxidant Activity

When evaluating the antioxidant activity by different techniques (Table 4), CFH showed 98.24 mM FeSO_4_/ g DE in the FRAP test, compared to the control, which showed a value of 75.50 mM of FeSO_4_/ g DE. In the TEAC test our extract (CFH) showed a value of 415.25 mg trolox/g DE and a value of IC_50_ of 0.051 mg/ml in the DPPH test, compared to the ascorbic acid used as control which showed a DPPH value of 0.045 mg/ml.

## 4. Discussion

Hyperglycemia is considered to be the main cause of micro- and macrovascular complications in diabetes mellitus; if the hyperglycemia persists, it causes the concentration of advanced glycation end products (AGEs) to increase. [21] This increase in the concentration of AGEs has been related to the aging process, as well as in the pathogenesis of age-related disorders, including Alzheimer's disease and diabetic complications [22].

The formation of AGEs starts from a series of complex reactions between the carbonyl of a reducing sugar or aldehyde and the free amino group of a protein, resulting in the reversible formation of Schiff bases. Molecular rearrangements then form covalently bound Amadori products, which induce further oxidation resulting in dicarbonyl compounds, forming fluorescent cross-links (e.g., pentosidine) in addition to nonfluorescent compounds (e.g., N^ɛ^-(carboxymethyl)lysine, CML), referred to as AGEs [23].

Aminoguanidine, a synthetic inhibitor of glycation, decreases the development of diabetic complications; however, toxic effects have been reported in clinical trials using this drug [24]. Therefore, the discovery of new inhibitors of the protein glycation reaction provides a promising therapeutic approach to prevent complications in diabetes [3]. Based on the fluorescence property, the effect of CFH on the formation of total AGEs in different periods of time (1, 2, 3, and 4 weeks) was evaluated. The results show that CFH effectively inhibits the glycation reaction; since the formation of advanced glycation end products is favored by oxidative reactions, CFH could inhibit its formation by reducing the reactive oxygen species (ROS) or by trapping the ROS formed in vitro by the autoxidation of sugars and/or the oxidative degradation of Amadori products such as fructosamine.

The formation of AGEs increases when the concentrations of oxoaldehydes and fructosamine rise [3]; in this regard our results indicate that CFH effectively reduces the concentration of fructosamine, which helps reduce some of the complications associated with AGEs.

The chemical modifications of proteins by carbohydrates during glycation modify their structure, function, and rotation, which leads to the deterioration of biological systems during aging. Maillard reactions are the main nonenzymatic pathways that contribute to protein damage, autoxidation of sugars, and oxidative degradation of Amadori products, increasing the chemical modifications of proteins by ROS [25, 26]. Amadori products can contribute to the development of diabetic complications through the oxidation of proteins induced by free radicals [27]. When proteins are oxidized, protein carbonyl is formed with the subsequent loss of protein thiols, which have been widely used as protein oxidation indexes [28]. When evaluating the obtained results it was observed that the BSA/glucose/CFH system reduced oxidative damage, possibly due to the decrease in the formation of ROS, protecting the thiol groups from oxidation. This indicates that CFH reduces oxidative stress and is effective in protecting proteins from oxidative damage, which occurs during the glycation process.

CML is formed by the oxidative decomposition of Amadori products and during the oxidation catalyzed by polyunsaturated fatty acids in the presence of proteins, it does not present fluorescence and is one of the main AGEs in vivo [29]; it is found predominantly in the kidneys of people with diabetes [30]. Our results indicate that CFH inhibits the formation of CML, which suggests that the hexane extract of* Spondias purpurea* is effective in reducing the complications of diabetes.

The antioxidant capacity of CFH was evaluated in vitro using the FRAP trial, which was used to evaluate the capacity to reduce iron ions, in order to determine the capacity to reduce ROS [31]. The TEAC trial was used to determine the ability of a sample to remove ABTS radicals [32]. DPPH is widely used to evaluate the ability of free radical scavenging of phytochemical compounds in vitro. CFH showed an effect in the reduction of free radicals in the DPPH test, as well as antioxidant activity in the FRAP test. The results suggest that CFH owes its antioxidant activity to its content of bioactive compounds with antioxidant properties, which act on harmful molecules such as free radicals, interacting and destabilizing proteins, nucleic acids, and lipids; however, other antioxidant compounds with properties may exist in the matrix of the sample, such as vitamins, enzymes, and minerals [9]; furthermore, the reducing capacity of ferric ions of CFH corroborates its antioxidant potential. CFH was able to inhibit the formation of AGEs by reducing the formation of ROS or by eliminating the ROS formed in vitro by means of the autoxidation of sugars and/or the oxidative degradation of the Amadori products.

## 5. Conclusions

The results obtained in this study show that the hexane extract of* Spondias purpurea *shows antioxidant activity and can inhibit the nonenzymatic glycation reaction of proteins, possibly by inhibiting oxidative processes. However, further studies are required to determine the mechanism by which CFH inhibits the glycation reaction, as well as additional studies to characterize its bioactive components responsible for the observed activities, which are ongoing.

## Figures and Tables

**Table 1:** Effect on fructosamine levels using CFH at different concentrations.

Experimental groups	Fructosamine (mM)			
	Week 1	Week 2	Week 3	Week 4
BSA/glucose	80.25±1.3	83.64±1.2	84.95±1.2^c^	87.25±0.5
BSA/glucose/CFH (0.30 mg/ml)	81.23±1.2	83.91±1.2^a^	86.21±1.2^a^	88.38±1.3^a^
BSA/glucose/CFH (0.60 mg/ml)	78.73±1.2^a^	82.14±1.2^b^	84.82±1.2^c^	86.94±1.5^d^
BSA/glucose/CFH (1.2 mg/ml)	76.54±1.0^a^	78.93±1.8^b^	81.02±0.5^c^	84.85±1.0^d^
BSA/glucose/CFH (2.5 mg/ml)	76.21±1.3^a^	78.85±0.9^b^	80.95±0.7^c^	82.44±0.8^d^
BSA/glucose/CFH (5 mg/ml)	75.71±1.2^a^	77.87±1.3^b^	79.54±1.6^c^	80.74±1.3^d^
BSA/glucose/AG (5 mg/ml)	75.32±1.2^a^	77.51±1.5^b^	78.12±1.3^c^	79.59±0.7^d^

Results are expressed as mean ± SEM (n = 3). ^a^p < 0.05 when compared to BSA/glucose at week 1; ^b^p < 0.05 when compared to BSA/glucose at week 2; ^c^p < 0.05 when compared to BSA/glucose at week 3; ^d^p < 0.05 when compared to BSA/fructose at week 4.

**Table 2:** Determination of the protein carbonyl content in the BSA/glucose system using CFH at different concentrations.

Experimental groups	Protein carbonyl content (nmol/mg protein)			
	Week 1	Week 2	Week 3	Week 4
BSA/glucose	80.12±1.8	82.19±1.4^a^	83.11±1.4^a^	84.09±1.2^a^
BSA/glucose/CFH (0.30 mg/ml)	78.88±0.7^a^	80.45±0.8^b^	82.87±1.1^c^	83.92±1.1^d^
BSA/glucose/CFH (0.60 mg/ml)	77.32±1.1^a^	78.85±1.7^b^	82.07±1.4^c^	82.97±1.1^d^
BSA/glucose/CFH (1.2 mg/ml)	76.02±9.9^a^	78.64±1.4^b^	81.89±1.2^c^	82.22±0.9^d^
BSA/glucose/CFH (2.5 mg/ml)	75.55±1.2^a^	77.67±0.8^b^	80.37±1.4^c^	82.11±1.1^d^
BSA/glucose/CFH (5 mg/ml)	74.80±0.6^a^	76.36±0.7^b^	79.08±0.7^c^	80.49±0.6^d^
BSA/glucose/AG (5 mg/ml)	73.65±1.7^a^	74.71±0.7^b^	77.91±0.3^c^	78.38±1.1^d^

Results are expressed as mean ± SEM (n = 3). ^a^p < 0.05 when compared to BSA/fructose at week 1; ^b^p < 0.05 when compared to BSA/fructose at week 2; ^c^p < 0.05 when compared to BSA/fructose at week 3; ^d^p < 0.05 when compared to BSA/fructose at week 4.

**Table 3:** Effects of CFH on the level of thiol group in BSA/glucose system.

Experimental groups	Thiol group (nmol/mg protein)			
	Week 1	Week 2	Week 3	Week 4
BSA/glucose	75.98±1.2	69.38±0.07^a^	64.88±0.05^a^	60.05±0.08^a^
BSA/glucose/CFH (0.30 mg/ml)	75.75±1.0	72.73±0.09^b^	68.16±0.07^c^	61.52±0.06
BSA/glucose/CFH (0.60 mg/ml)	75.38±0.51	73.25±0.12^b^	68.49±0.08^c^	66.43±0.04^d^
BSA/glucose/CFH (1.2 mg/ml)	77.18±0.83^a^	73.54±0.09^b^	69.36±0.11^c^	67.33±0.09^d^
BSA/glucose/CFH (2.5 mg/ml)	76.41±0.11^a^	74.38±0.07^b^	69.68±0.08^c^	67.66±0.04^d^
BSA/glucose/CFH (5 mg/ml)	78.30±0.08^a^	75.08±0.05^b^	70.61±0.09^c^	68.52±0.06^d^
BSA/glucose/AG (5 mg/ml)	79.52±0.08^a^	75.47±0.02^b^	71.56±0.04^c^	69.08±0.09^d^

Results are expressed as mean ± SEM (n = 3). ^a^p < 0.05 when compared to BSA/fructose at week 1; ^b^p < 0.05 when compared to BSA/fructose at week 2; ^c^p < 0.05 when compared to BSA/fructose at week 3; ^d^p < 0.05 when compared to BSA/fructose at week 4.

**Table 4:** CFH's antioxidant activity.

	FRAP (mM de FeSO_4_/ g DE)	DPPH (IC_50_) (mg/ml)	TEAC (mg trolox/g DE)
**Ascorbic acid**	75.50±1.58	0.045±2.1	-
**CFH**	98.24±1.02	0.051±1.6	415.25±1.4

Values are expressed as mean ± SD (n=3) and DE (dry extract)

## Data Availability

The data used to support the findings of this study are included within the article.
